# Supplementary material for: BayesR3 enables fast MCMC blocked processing for largescale multi-trait genomic prediction and QTN mapping analysis
Source: Commun Biol. 2022 Jul 5;5:661. doi: 10.1038/s42003-022-03624-1 (PMC9256732; doi:10.1038/s42003-022-03624-1)
Supplement: Supplementary file 2 — Supplementary Information [file 42003_2022_3624_MOESM2_ESM.pdf]

BayesR3 enables fast MCMC blocked processing for largescale multi-trait genomic prediction and QTN mapping analysis

Edmond J. Breen[1], Iona M. MacLeod[1], Phuong N. Ho[1], Mekonnen Haile-Mariam[1], Jennie E. Pryce[1,2], Carl D. Thomas[1], Hans D. Daetwyler[1,2], and Michael E. Goddard[1,3]

[1] Agriculture Victoria, AgriBio, Centre for AgriBioscience, Bundoora, Victoria 3083, Australia
[2] School of Applied Systems Biology, La Trobe University, Bundoora, Victoria 3083, Australia
[3] Faculty of Veterinary and Agricultural Sciences, University of Melbourne, Parkville, VIC, 3052 Australia

Supplementary Note 1

Mixture Model Distribution

The mathematics used for BayesR3 is given in detail in the methods section of the main manuscript, but briefly the SNP effects are modelled by a mixture of four normal distributions with zero mean and increasing variances as specified by:

$$p\left(g_j \middle| \pi, \sigma_g^2\right) = \pi_1 \times N\left(0, 0 \times \sigma_g^2\right) + \pi_2 \times N\left(0, 10^{-4} \times \sigma_g^2\right) + \pi_3 \times N\left(0, 10^{-3} \times \sigma_g^2\right) + \pi_4 \times N\left(0, 10^{-2} \times \sigma_g^2\right) \quad (1)$$

Where σ_g^2 is the additive genetic variance explained by the SNPs cumulatively and is estimated from the data. The mixing proportions π are also estimated from the data and are assumed to be drawn from a Dirichlet distribution with parameter = (1,1,1,1), a uniform prior, such that any SNP a priori is equally likely to be assigned to any one of the 4 distributions. The choice of 4 distributions, is historical (1), but any number of distributions can be used. For example, in very large datasets, adding the variance group $10^{-5} \times \sigma_g^2$ can help capture SNPs with very small effects (2). However, the allocations values $(0, 10^{-4}, 10^{-3}, 10^{-2})$ seen in Equation (1) can mimic a broad range of parametric distributions, such as a t or a reflected gamma, by varying the proportions π in each distribution. They can describe a distribution with long tails as we expect for SNP effects where there are many small effects and the occasional large effect (Figure S1).

The 10x scaling between the allocation values is arbitrary but convenient in practice. It allows the distributions generated to be relatively smooth and effects can shuffle from one distribution to the next between MCMC cycles. Figure S1 shows a distribution that is a mixture of 3 normal distributions with variances (0.0001, 0,001 and 0.01) in blue compared to a mixture of 6 distribution with variances 0.00005, 0.0001, 0.0005, 0.001, 0.005, 0.01 in red. They are very similar and the use of either one as the prior would have little effect on the resulting estimated SNP effects.

These allocation variances could be estimated from the data, but this is unnecessary and introduces additional complexity. The fact that similar distributions can be generated by different mixtures is a warning that the data cannot distinguish a variety of possible distributions because they are essentially the same. Also, if the variances were sampled within the MCMC process the large variance and small variance would at times swop, otherwise the chain is not mixing fully. This makes it difficult to interpret the summary statistics from the chain.

Therefore, we think that allowing the proportions π and σ_g^2 to be derived from the data gives the model ample flexibility to fit a variety of useful distributions. It is also worth noting that Bayes R has been published many times with this arrangement of variances.

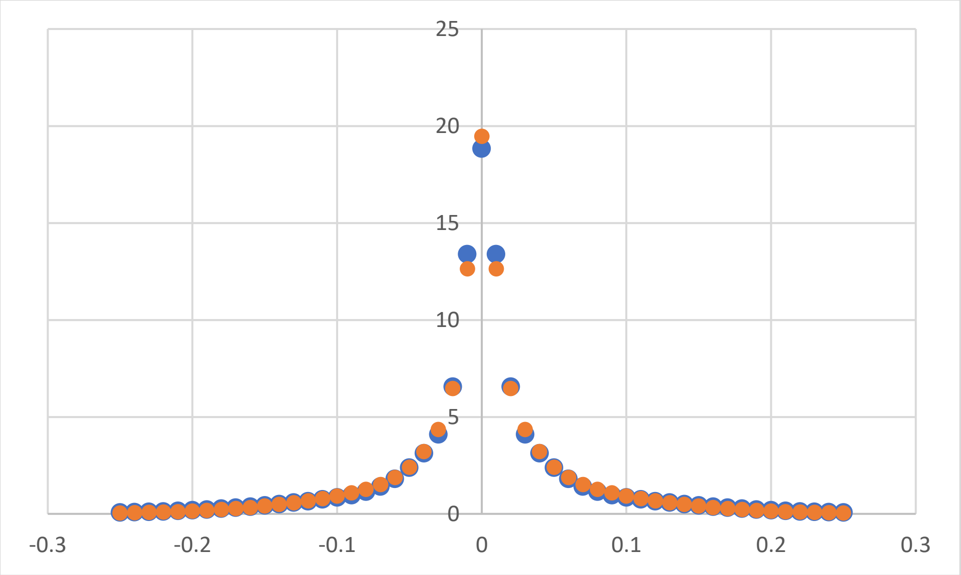

Figure S1: Mixture distributions. A distribution made up of equal parts of 0.0001, 0.001 and 0.01 variances (blue dots) and a 2nd mixture distribution made from 6 normal distributions with variances 0.00005, 0.0001, 0.0005, 0.001, 0.005, 0.01 in proportions 0.11, 0.16, 0.16, 0.17, 0.17, 0.23 in orange.

Supplementary References

1. Erbe M, Hayes BJ, Matukumalli LK, Goswami S, Bowman PJ, Reich CM, et al. Improving accuracy of genomic predictions within and between dairy cattle breeds with imputed high-density single nucleotide polymorphism panels. J Dairy Sci. 2012;95(7):4114-29.
2. Moser G, Lee SH, Hayes BJ, Goddard ME, Wray NR, Visscher PM. Simultaneous discovery, estimation and prediction analysis of complex traits using a bayesian mixture model. PLoS Genet. 2015;11(4):e1004969.

Supplementary Note 2

Determining an Optimal Block Size

Here we look at two methods for determining the optimal block size and one method to determining the number of inner iterations.

Optimizing Block Size and the Number of Inner Cycles by Constraining Accuracy and Minimizing Time.

In determining the optimal block size, it is noted with respect to a given chain length, that as block size increases BayesR3 predication accuracy decreases, albeit only slightly, (see Figure 4 main manuscript), as processing time rapidly decreases. This occurs, until a certain block size is reached, after which processing time remains essentially constant and at times may even increase as block size increases. Therefore, we looked for a simple method to define in generally terms the optimal block size and number of inner iterations.

Let

$$n_M = \text{No. of SNPs}$$
$$n = \text{No. of SNPs per block}$$
$$\frac{n_M}{n} = \text{No. of blocks}$$
$$n_R = \text{No. of Records}$$
$$x = \text{No. of inner cycles}$$
$$y = \text{No. of outer cycles}$$
$$xy = \text{total no. of cycles}$$

The number of blocks is determined by the smallest integer larger or equal to the ratio $\frac{n_M}{n}$, this means that all blocks will be the same size except for the last block, which can be smaller.

Time taken:

The major contributors to the processing time are:

1. Calculating the sum for each SNP when processing a new block. This is proportional to: $yn_M n_R$.
2. Cycling around a block, sampling a SNP effect, and updating the total for all other SNPs in the block. This is proportional to $yn_M xn$

Then:

$$\text{Time} = yn_M n_R + yn_M xn$$

Accuracy:

We wish to take enough samples of each SNP effect to reduce the Monte Carlo sampling error of the mean to an acceptably low value. Each individual sampled value of a SNP effect $(\hat{b})$ can be modelled as:

$$\hat{b}_{ij} = b + u_i + e_{ij}$$

where $\hat{b}_{ij}$ is the sampled value of b in outer cycle i and inner cycle j. u_i = effect common to all samples in outer cycle i, and e_{ij} is the effect of the inner cycle j within outer cycle i.

The variation of the samples within the same block are correlated because they are based on the sampled values from all other blocks. This correlation is the reason why u_i is included in the above formula for $\hat{b}_{ij}$. The importance of u_i depends on the LD between SNPs in the current block and SNPs in all other blocks. This LD decreases as block size increases. Therefore, we will assume:

$$v(u_i) \propto \frac{1}{n}$$

$$v(\hat{b}_{ij}) = \frac{\sigma^2}{n} + \sigma^2$$

and the Monte Carlo variance of the average $\hat{b}_{ij}$ is: $\frac{\sigma^2}{yn} + \frac{\sigma^2}{xy}$.

We wish to optimize x and n by minimizing the time taken while holding constant the Monte Carlo sampling variance. Using a Lagrange multiplier, the objective is:

$$y n_M (n_R + xn) + \lambda\left(\frac{1}{ny} + \frac{1}{xy} - k\right)$$

$$\frac{\delta}{\delta n} = y n_M x - \lambda \frac{1}{n^2 y} = 0 \Rightarrow n^2 = \frac{\lambda}{n_M x y^2}$$

$$\frac{\delta}{\delta x} = y n_M n - \lambda \frac{1}{x^2 y} = 0 \Rightarrow x^2 = \frac{\lambda}{n_M n y^2}$$

Therefore, $n = x$ and the constraint becomes $\frac{2}{ny} - k = 0$, and hence

$$y = \frac{2}{nk}$$

$$\begin{aligned}
\text{Time} &= y n_M (n_R + xn) \\
&= \frac{2 n_M}{nk}(n_R + n^2) \\
&= \frac{2 n_M}{k}\left(\frac{n_R}{n} + n\right) \\
\frac{\delta}{\delta n} &= \frac{2 n_M}{k}\left(\frac{-n_R}{n^2} + 1\right) = 0 \\
&\Rightarrow n = \sqrt{n_R}
\end{aligned}$$

Thus, the approximate optimum is $n = x = \sqrt{n_R}$. This is approximate because of the assumptions made and the exact optimum may be data set dependent. However, it is intuitively reasonable: If there are many SNPs per block, the value for each SNP depends on the current value if all the other SNPs in the block so it is worthwhile to take many cycles around the block. The time saved by blocking is due to processing the individual records only once per block and therefore as n_R increases the optimum n also increases.

Determining the optimal block size using a curvature equation

Given the number of inner iterations is set to equal the number of SNPs within a block, we note the observed change in processing time with respect to block size for a given genomic data set as shown in Figure 5b (main manuscript), was successfully model using the function $f(n) = \frac{n_R + n}{n}$. This function has one point on the curve where the curvature is a maximum, and which corresponds to a transition from high to low curvature. The derivative of $f(n)$ is $f'(n) = \frac{-n_R}{n^2}$, $f''(n) = \frac{2n_R}{n^3}$, and the curvature of the curve, in Figure 5c main manuscript, is given by $\kappa(n) = \left|\frac{f''(n)}{(1+(f'(n))^2)^{\frac{3}{2}}}\right|$. When the derivative of κ is set to zero, it has a corresponding positive root where the curvature is maximized and where $n = \sqrt{n_R}$. In terms of optimization this represents an elbow or knee point and is the optimum between the benefit achieved between a reduced processing time and the loss in

prediction accuracy as n increases. Therefore, we suggest that the block size should not increase beyond $\sqrt{n_R}$.

Supplementary Tables

Supplementary Table 1: Details of the QTL annotated in ***Error! Reference source not found.*** *for milk composition traits discovered in the multi-trait BayesR3 MIR analysis as well as the multi-trait Milk, Fat and Protein Yield BayesR3 (MFP_BR3) and BayesR3C (MFP_BR3C) analyses. Details include the underlying candidate genes and previously reported overlapping milk trait QTL.*

**QTL midpoint position (bp: see** Error! Reference source not found.**)**	**Multi-trait analysis detecting the QTL**	**Candidate gene(s) and start…stop position (bp)**	**Examples of published reports for milk traits**	**Examples of milk traits previously reported for this QTL position**
Chr1:142827704	MIR	*SLC37A1* 142772300…142873917	(30, 41)	P, Milk Yield<br><br>P, Mg, K, Na
Chr2:131204809	MIR	*ALPL* 131181421..131268191	(30)	Na
Chr3:15387272-15484820	MIR<br>MFP_BR3<br>MFP_BR3C	*SLC50A1* 15463149…15465593<br>*DPM3* 15462231...15462806<br>*EFNA1* 15466565…15473164<br>*LOC107132270* (ncRNA) 15465686…15496399	(30, 31)	Lactose percentage<br><br>Na
Chr5:93534138-93538860	MIR<br>MFP_BR3<br>MFP_BR3C	*MGST1* 93495438…93520998<br>*SLC15A5* 93602194…93699207	(25, 31, 42)	fat percentage<br><br>Lactose yield<br><br>Milk and Fat yield, Fat percentage
Chr6:45052030	MIR	*LOC112447058* ncRNA 45060561…45065030	(30)	P, K
Chr6:85419916-85475175	MIR<br>MFP_BR3<br>MFP_BR3C	*CSN2* 85449173...85457867<br>*CSN1S1* 85411601…85429256	(25, 30, 41)	protein yield, protein percent<br><br>Na, Ca<br><br>Milk and Protein Yield
Chr11:103243985	MIR<br>MFP_BR3<br>MFP_BR3C	*PAEP* 103255963…103260862	(30, 41, 43)	Milk, fat and Protein Yield, Fat percentage Protein percentage<br><br>Citrate, P
Chr11:104186080	MFP_BR3<br>MFP_BR3C	*ABO* 104176830…104214758	(25, 44)	Protein yield<br><br>oligosaccharides
Chr14: 263754 - 792410	MIR<br>MFP_BR3<br>MFP_BR3C	*DGAT1* 603981…614153	(29, 45)	Milk, fat and protein yield, fat and protein percentage

		(many other genes including solute carriers *SLC39A4* and *SLC52A2*)		
Chr15:81098284	MFP_BR3<br>MFP_BR3C	*CTNND1* and olfactory receptor genes	No reports found	-
Chr16:1750054 - 1803090	MFP_BR3<br>MFP_BR3C	*SOX13* 1867662...1912526<br>*LOC104974354* 1791622...1806028	No reports found	-
Chr20:31887560	MFP_BR3<br>MFP_BR3C	*GHR*<br>31869704…32043372	(43, 46, 47)	Milk, Fat and Protein Yield, Fat and Protein percentage, Milk Yield<br><br>Protein percent
Chr20:58389561	MIR<br>MFP_BR3<br>MFP_BR3C	*ANKH*<br>58307527…58477499	(30, 31, 41)	Fat yield, protein percentage<br><br>Lactose percentage<br><br>K
Chr27:36499460	MIR	*GPAT4* (*AGPAT6*)<br>36508780…36539760	(30, 48)	Fat and protein percentage, protein yield, lactose yield and percentage, milk fatty acids<br>Mg

Supplementary Table 2: Distribution of SNP effects on PCs derived from milk, fat, and protein yield and PC heritabilities. Note the loadings give the coefficients of the linear combinations of the centered and scaled continuous variables for milk, fat, and protein yield. Distributions 1-4 are each normal distributions with mean = 0 and variances = 0, 0.0001, 0.001, 0.01 times the genetic variance, respectively.

**PC**	**PC loadings**			**Distribution**				h^2
	Milk	Fat	Protein	k_1	k_2	k_3	k_4	
1	-0.603	-0.493	0.627	140	9801	5	2	0.42
2	-0.443	0.861	0.251	2688	7219	12	30	0.48
3	-0.663	-0.126	-0.738	2293	7555	79	21	0.52

Supplementary Table 3: Distribution of SNP effects on PCs derived from milk, fat and protein yield when using prior information from analysis of milk MIR spectra.

Class	Total Number of SNPs	Number of SNPs in Model	PC	Distribution			
				k_1	k_2	k_3	k_4
1	992	48 (4.8%)	1	7	30	9	2
			2	5	4	27	11
			3	2	2	32	12
2	238677	4084 (1.6%)	1	138	3940	5	1
			2	614	3460	8	12
			3	348	3660	58	5
3	238667	4061 (1.6%)	1	438	3520	3	0
			2	1880	2100	4	4
			3	1340	2640	5	3
4	157535	2665 (1.5%)	1	202	2400	3	0
			2	1140	1440	2	0
			3	1560	1036	2	0

Supplementary Table 4: Multi-Trait Analysis of Milk Production Traits of Dairy Cattle – accuracy of prediction using BayesR3 and BayesR3C. BayesR3C used the multi-trait MIR Q2 probabilities to allocate variants to four classes (see Materials & Methods in full paper). Reference N=65,637, & Validation populations were as described for the Single Trait Analyses: HOL_Bull was 702 Holstein bulls, JER_Bull was 675 Jersey bulls and RDC_Cows included 3082 Australian Red cows. Accuracy was averaged across 5 MCMC chains for each PC trait.

Validation Set	PC Trait	BayesR3 Accuracy	BayesR3C Accuracy
Hol_Bull	1	0.708	0.710
	2	0.819	0.818
	3	0.841	0.840
JER_Bull	1	0.753	0.753
	2	0.799	0.800
	3	0.852	0.854
RDC_Cows	1	0.235	0.231
	2	0.396	0.384
	3	0.255	0.263
