# Supplementary material for: BayesR3 enables fast MCMC blocked processing for largescale multi-trait genomic prediction and QTN mapping analysis
Source: Commun Biol. 2022 Jul 5;5:661. doi: 10.1038/s42003-022-03624-1 (PMC9256732; doi:10.1038/s42003-022-03624-1)
Supplement: Supplementary file 3 — Description of Additional Supplementary Files [file 42003_2022_3624_MOESM3_ESM.pdf]

Description of Additional Supplementary Files

File name: Supplementary Data 1
Description: Data file giving the top 1000 SNPs and other selected genotypes and SNP rankings for various analysis with respect to milk production traits and MIR data

File name: Supplementary Data 2
Description: The source data behind the manuscript figures

File name: Supplementary Software 1
Description: R source code for single trait bayesR3
